# China economy-wide material flow account database from 1990 to 2020

**DOI:** 10.1038/s41597-022-01611-z

**Published:** 2022-08-17

**Authors:** Chuke Chen, Jianchuan Qi, Nan Li, Tiantian Ji, Heming Wang, Yuanyi Huang, Jing Guo, Xiaohui Lu, Ruru Han, Jianlimin Wei, Wei-Qiang Chen

**Affiliations:** 1 Key Lab of Urban Environment and Health, Institute of Urban Environment, Chinese Academy of Sciences, Xiamen, 361021 P. R. China; 2 Xiamen Key Lab of Urban Metabolism, Xiamen, 361021 P. R. China; 3 University of Chinese Academy of Sciences, Beijing, 100049 P. R. China; 4 Ganjiang Innovation Academy, Chinese Academy of Sciences, Ganzhou, 341119 P. R. China; 5 State Environmental Protection Key Laboratory of Eco-Industry, Northeastern University, Shenyang, 110819 P. R. China; 6 College of Civil and Transportation Engineering, Shenzhen University, Shenzhen, 518060 P. R. China; 7 Nottingham University Business School, University of Nottingham Ningbo China, Ningbo, 315100 P. R. China

**Keywords:** Environmental economics, Sustainability

## Abstract

Material utilisation has been playing a fundamental role in economic development, but meanwhile, it may have environmental and social consequences. Given China’s rapid economic growth, understanding China’s material utilisation patterns would inform decisions for researchers and policymakers. However, fragmented data from multiple statistical sources hinder us from comprehensively portraying China’s material utilisation dynamics. This study harmonised China-specific official statistics and constructed a China economy-wide material flow accounts database. This database covers hundreds of materials and more than 30 years (1990–2020) from thousands of data sources, which is comprehensive, long-term, up-to-date, and publicly accessed. This database would provide insights into the historical metabolic dynamics of China’s economy with elaboration on the production, consumption, and end-of-life disposal of materials. This database also allows for international analyses since it is developed based on an internationally standardised analytical framework. Furthermore, this study would benefit studies on policy impact evaluation, environmental pressure assessment, and sustainable development strategies.

## Abstract (summary)

**Table:** 

Measurement(s)	domestic extraction • emission • international trade • imports • exports
Technology Type(s)	economy-wide material flow analysis
Factor Type(s)	economy • environment • material flow
Sample Characteristic - Location	China

## Background & Summary

Materials significantly contribute to sustainable development in a direct and indirect way^1^. That is, the utilisation of materials has been playing a fundamental role in economic growth and well-being, but at the same time, it may have environmental and social consequences. China has been rapidly developing with a massive number of materials utilised. Understanding China’s historical material utilisation patterns are essential for formulating appropriate policies for achieving global Sustainable Development Goals (SDGs)^2^, which necessitates a detailed quantitative picture of material production, consumption, and end-of-life disposal.

There have been several attempts to explore national material utilisation. Some studies focused on developing and improving the statistical accounting framework for quantifying material utilisation in terms of flows. The economy-wide material flow analysis (EW-MFA) is the origin and most widely used^3^, which depicts how the national economy system receives external materials and energy (i.e., input) from and releases wastes (i.e., output) to the environment. This framework was initially established in 2000 by the European Commission Statistical System (Eurostat) and was improved and adopted as an internationally standardised environmental-economic system accounting method by the Statistical Commission of the United Nations (UN) in 2012. The latest version of this framework was released in 2018^4^. Based on this analytical framework, several EW-MFA indicators were derived to depict the metabolic processes^5^, which aid in developing effective strategies^6^. For example, the SDG targets for resource productivity (SDG 8.4) and sustainable use of natural resources (SDG 12.2) can be evaluated with EW-MFA results of the domestic material consumption. Previous EW-MFA studies on China’s economy revealed amounts, components, efficiencies, etc. of material utilisation for particular purposes, such as, monitoring the progress of the circular economy between 1995 and 2015^7^, analysing policy effects between 1990 and 2002 for sustainable development^5^ and between 2000 and 2010 for circular economy^8^, formulating the 13^th^ Five-Year plan strategies (2016–2020) based on historical patterns from 1992 to 2014^9^. However, a long time-series profile of China’s rapid growth since the 1990s has yet to be identified.

Other studies have focused on harmonising data compilation procedures for EW-MFA application, which are essential for comparative analyses across multiple nations and periods. Typically, estimates of national material utilisation are based on a variety of data sources, which were gathered using various statistical criteria. For instance, agricultural statistics from the UN Food and Agriculture Organization (FAO)^10^, Eurostat foreign trade statistics, the United States geological survey (USGS), etc., were used in the EW-MFA of the EU. Before being applied to estimates, these original statistics need to be adjusted and aggregated. However, due to an incomplete understanding of data gathering and pre-processing procedures, uncertainties and biases may exist in further data interpretation^3^. For example, the USGS uses the weight unit of metal content to record the quantity of mineral production, whereas the China statistical department uses the gross ore unit, resulting in considerable differences in values. So far, existing data compilation tools are country/area-specific (e.g., for the EU^4,11^, the Asia-Pacific region^12^) or year/period-specific (e.g., 1999^13^, 2000^14^, and 20 century^15^), which are inapplicable to the China’s statistics. In addition to the differences between multinational datasets, uncertainties may arise, for example, from inevitable artificial errors during data preparation. However, it is time-consuming and labour-intensive to assess reliability related to data collection procedures since original statistics are fragmented over multiple databases and have to be processed before applications.

In this study, we first identify system boundaries, material types, materials, and processes related to China’s economy based on the internationally-standardised analytical framework (Economy-Wide Material Flow Analysis, EW-MFA), examine the reporting standards, material coverages, and time periods of official China-specific statistics, apply them to characterise China’s material utilisation patterns between 1990 and 2020, and analyse uncertainties from data collection and estimation procedures. This dataset covers hundreds of materials that have been extracted, emitted, imported, exported, and stored in China for more than thirty years. This dataset can be applied to the studies, including but not limited to:

- Quantifying the in-use material stocks to evaluate the progress of the circular economy.
- Analysing the sustainability of material use for any substances or materials in China to evaluate the progress toward the SDGs.
- Compilating the Physical Input-Output Tables (PIOT) to aid in the analysis of the effects of policy on specific economic activities.

## Methods

### China economy-wide material flow identification: system boundary, processes, and materials

The first step is to define an economy, i.e., the economic (rather than geographical) territory of a country in which the activities and transactions of producer and consumer units are resident. Additionally, the period is a total of thirty-one years, from 1990 to 2020, for the following reasons: (1) statistics before 1990 are of poor quality and are insufficient to allow us to conduct analyses; and (2) so far, statistics have just recently been updated to cover the year of 2020. Furthermore, the analytical framework (hereinafter referred to as China EW-MFA) is developed to explore material utilisation and its environmental consequences within China’s economy.

**Fig. 1:**
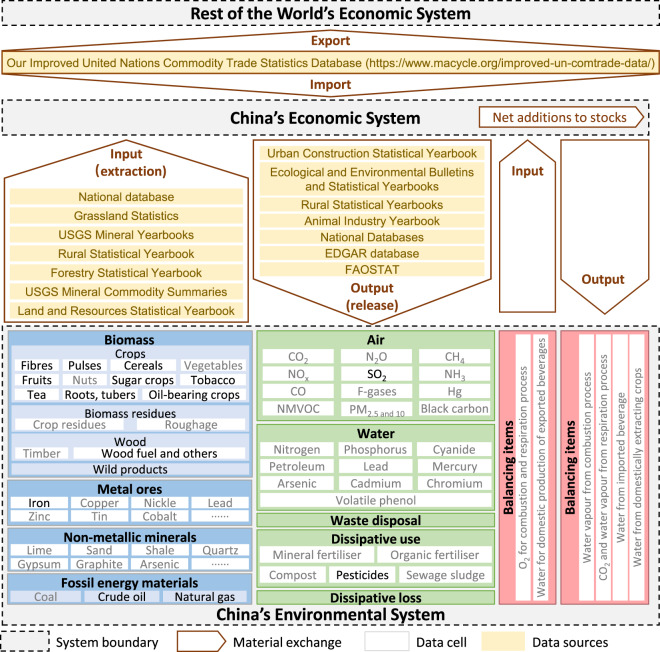
The general structure of China EW-MFA. To note, white data cells can be obtained directly from official statistics, whereas grey cells are estimated.

The general structure of China EW-MFA is depicted in Fig. 1, which comprises seven processes. **(1) Input of extracted resources:** domestic natural resources are extracted from the environment to the economy through human-controlled means. **(2) Output of domestic processed materials**: after being processed by manufacturers, materials are released from the economy into the environment in the form of by-products and residues, which can be classified by their destinations (i.e., air, land, and water) and pathways (dissipative use and losses). **(3) Input and (4) output by cross-border trade:** by imports and exports, materials are transported between China’s economy and the economies of the rest of the world. **(5) Input and (6) output of balancing items (*BI*):** sometimes, materials identified in the output processes are not considered by inputs, which needs to be balanced. For example, the utilisation of fossil energy materials by combustion causes the emission of carbon dioxide (CO_2_) into the air, which is identified as system output, but requirements of oxygen (O_2_) as system input are not counted. **(7) Additions to the system:** within the economy, materials would have been added to the economy in the form of buildings, infrastructures, durable goods, and household appliances, which are referred to as the net additions to stock (***NAS***).

The last step is to specify the materials concerned in each process. Four types (in blue boxes in Fig. 1) of natural materials are extracted and input into the economy in China, i.e., harvested biomass (33 items), mined metal ores (28 items), quarried non-metallic minerals (155 items), and mined fossil energy materials (6 items in 3 classes). Materials (green boxes) released into the air are greenhouse gases (e.g., CO_2_, methane (CH_4_), dinitrogen oxide (N_2_O)), air pollutants (e.g., particulate matter 10 (PM_10_), black carbon (BC)), and toxic contaminants of mercury (Hg) in divalent, gaseous elemental, and particulate forms. Those released into the water are inorganic matters (of nitrogen (N), phosphorus (P), Arsenic (As), and four heavy metals of lead (Pb), mercury (Hg), cadmium (Cd), and chromium (Cr)) and organic matters of cyanide, petroleum, and volatile phenol. Materials released into the land are waste disposal in uncontrolled landfills, which are illegal in China. Some materials are dissipated by application, for example, fertilisers, compost, sewage sludge being applied to agricultural land, and pesticides being used to cultivate crops. Some would be unintentionally dissipated from abrasion, corrosion, erosion, and leakages. Materials (in red boxes) are ***BI***, which includes the input of O_2_ and output of water vapour in the fossil energy material combustion process, the input of O_2_ and output of water vapour and CO_2_ in the respiration process of human and cultivated livestock, input and output of water in imported and exported beverages, and the output of water from domestically extracting crops.

There are some messages needed to be mentioned: (1) Material of water is not included since its flow volume is more substantial than others, which needs to be independently analysed; (2) Activities of foreign tourists, cross-border transfer of emissions through natural media, etc. are excluded. (3) To be clear, we refer to a data cell as a specific flow process of a specific substance in a specific year, e.g., the number of cereals domestically extracted in 2020.

### Data acquisition: sources and collection

Based on our China EW-MFA, we first analyse accessibility, reliability, completeness, rules of redistribution, etc., for each data source (yellow boxes in Fig. 1), including China national database, China rural statistical yearbooks, USGS mineral yearbooks, etc. The complete list of data sources and descriptions are presented in Table 1. Then, we store the originally retrieved data source files in a semi- or unstructured format (e.g., CSV, PDF). Next, we manually collect these statistics and reorganise them according to China EW-MFA material types and processes. However, only a tiny part of retrieved statistics can be applied directly, as specified in black colour in Fig. 1.

**Table 1:** Data sources and descriptions.

Data sources		Descriptions
1	China National database^16^	It covers twenty-eight themes (e.g., agriculture, resources, environment, industry, construction), derived indicators, and the period of 1978–2021 at many regional scales, maintained by the China National Bureau of Statistics.
2	China Rural Statistical yearbooks^40^	It covers twelve themes (e.g., agricultural products’ harvest, consumption, etc.), derived indicators, and the period of 1985–2021 at many regional scales, maintained by the China National Bureau of Statistics.
3	China Forestry Statistical Yearbooks^41^	It covers domestic production, consumption, investments, etc., of primary forest products from 1998 to 2019, maintained by the China National Forestry and Grassland Administration.
4	China Grassland Statistics^19^	It covers the production and consumption of seeds, grass products, etc., and natural disasters for grass industries from 2006 to 2010, maintained by the China Ministry of Agriculture and Rural Affairs.
5	China Land and Resources Statistical Yearbooks^20^	It presents the status of exploration, mining, consumption, etc., on natural resources and other related topics from 1999 to 2018, maintained by the Ministry of Land and Resources of China.
6	China Animal Industry Yearbooks^42^	It covers statistics related to the livestock, including, e.g., year-end, slaughtered, and sold numbers from 1999 to 2017, maintained by the China Ministry of Agriculture and Rural Affairs.
7	China Urban Construction Statistical Yearbooks^29^	It covers statistics related to urban management on waste, transportation, energy, etc., from 2002 to 2020 for many regional scales, maintained by the China Ministry of Housing and Urban-Rural Development.
8	China Ecological and Environmental Bulletins^22^ and Statistical Yearbooks^23^	It covers statistics related to sources, emissions, treatments, etc., of pollutants in the environment in China from 1989 to 2020, maintained by the China Ministry of Ecology and Environment.
9	Emissions Database for Global Atmospheric Research (EDGAR)^24^	EDGAR is a global database for anthropogenic emissions of greenhouse gases and air pollution on earth, developed under the United Nations Framework Convention on Climate Change (UNFCCC) using a consistent IPCC methodology.
10	FAOSTAT^10^	It covers thirteen domains of, e.g., production, food security and nutrition, and forestry, for over 245 countries and areas related to food and agriculture from 1961 to the most recent year available, maintained by the Food and Agriculture Organization (FAO) of the United Nations (UN).
11	The United Nations Commodity Trade Statistics Database (UN Comtrade)^31^	Maintained by the UN, it contains a repository of global trade data, including the year, value, quantity, and weight of imported or exported commodities and covers 209 countries or areas’ annual statistics since 1962 and monthly statistics since 2000. For the commodity classifications of Harmonised System published in 1992 (HS0), a total of 5,039 types of commodities are included. However, data quality issues are identified in UN Comtrade, which would result in significant uncertainties in analyses. Here, we used improved UN Comtrade data with data quality issues addressed by our previous attempts, and the dataset can be accessed online^32^.
12	USGS Minerals Yearbooks^21^	Published annually and maintained by the United States Geological Survey (USGS), it contains estimates covering nonfuel mineral industry structure, Government programs, tariffs, and 5-year salient statistics for about 90 individual mineral commodities from 1932 to 2022.
13	USGS Mineral Commodity Summaries^21^	

### Data compilation: parameter localisation and data estimation

A few inconsistencies in statistics were noticed, which would result in data incompleteness. For example, the domestic extraction of vegetables has been accounted for and published since 1995, before which statistics are unavailable. The domestically harvested timber has been measured in the volume unit of cubic metres, which needs to be converted into the mass unit via density conversion factor. Therefore, acquired statistics have to be estimated, which are specified in grey colour in Fig. 1. The following section elaborates on each data cell’s estimation methods, localised parameters, references, etc. In our uploaded data files, the original statistics, data sources, and compilation methods (using formulas) are all implemented, as explained in the Data Records Section.

- **The input of natural resources by domestic extraction**
  - **Vegetables in crops:** Statistics of vegetable production (*W*_*Vegetables*_)^16^ during 1990–1994 are unavailable, which is estimated based on the relationship between the production yield (*P*_*Yield*_) and areas (*A*_*Vegetables*_), as shown in Eq. 1. Here, *P*_*Yield*_ is assumed to remain constant at 27.04 thousand tonnes per thousand hectares from 1990 to 1995, derived by dividing vegetable production (257,267 thousand tonnes) by areas (9,515 thousand hectares) in 1995.
    1 $${W}_{Vegetables}={P}_{Yield}\times {A}_{Vegetables}$$
  - **Nuts in crops:** One of them is chestnuts. The chestnut production in 2020 is unavailable, which is assumed to be the same as in 2019.
  - **Crop residues in biomass residues:** They are referred to as that harvested production of crops that do not reach the market to be sold but are instead employed as raw materials for commercial purposes such as energy generation and livestock husbandry. This number (*W*_*crop residues*_) can be calculated by first determining the number of crop residues available from primary crop production (*W*_*crop*_) and the harvest factor (*P*_*harvest factor*_), and then using the recovery rate (*P*_*recovery rate*_) to determine the number of crop residues used by the economy, as shown in Eq. 2. These parameters have been localized by previous studies^17,18^, which are adopted in this study, i.e., wheat (1.1 for *P*_*harvest factor*_ and 0.463 for *P*_*recovery rate*_), maize (1.2, 0.463), rice (0.9, 0.463), sugar cane (0.5, 0.9), beetroots (0.7, 0.9), tuber (0.5, 0.463), pulse (1.2, 0.7), cotton (3.4, 0.463), fibre crops (1.8, 0.463), silkworm cocoons (1.8, 0.463), and oil-bearing crops (1.8, 0.463).
    2 $${W}_{cropresidues}={W}_{crop}\times {P}_{harvestfactor}\times {P}_{recoveryrate}$$
  - **Roughage of grazed biomass and fodder crops in biomass residues:** In China, the grazed **biomass** for roughage includes annual forage and perennial forage, whereas fodder crops comprise straw feed, processed straw feed, and all other fodder crops. However, information^19^ on grazed biomass production is only accessible from 2006 to 2018, whereas fodder crop statistics are only available from 2015 to 2017. Equation 3 and Eq. 4 can be used to estimate unavailable statistics. To note, we assume that China’s domestic roughage supply structure has remained unaltered, which has two meanings. The proportion of total domestic roughage production (*W*_*Domestic production*_) in requirement (*W*_*Roughage requirement*_) has remained constant, while the proportion (*P*_*Supply fraction*_) of grazed biomass and fodder crop in domestic roughage production has been unchanged. The requirement (*W*_*Roughage requirement*_) is determined by the quantity of livestock (*Q*_*Livestock*_) and their annual feeding amount (*P*_*Annual intake*_). *P*_*Annual intake*_ (in tonnes per head per year) has been localised for each type of livestock^4^, with 4.5 for live cattle and buffaloes, 0.5 for sheep and goats, 3.7 for horses, and 2.2 for mules and asses.
    3 $${W}_{Roughagerequirement}={Q}_{Livestock}\times {P}_{Annualintake}$$
    4 $${W}_{Domesticproduction}={W}_{Roughagerequirement}\times {P}_{Supplyfraction}$$
  - **Timber in wood:** As illustrated in Eq. 5, wood production^16^ is reported in volume units of cubic **metres** (*V*_*Timber*_), which need to be converted into mass units (*W*_*Timber*_) via density (*P*_*Density*_). The parameter *P*_*Density*_ is assumed to be 0.58 tonnes per cubic metre, calculated by averaging 0.52 for coniferous types and 0.64 for non-coniferous ones^4^.
    5 $${W}_{Timber}={V}_{Timber}\times {P}_{Density}$$
  - **Non-ferrous metals in metal ores:** Non-ferrous metal statistics are derived from two sources. China statistics^20^ are measured in gross ore (*W*_*Metal ores in gross ore*_) but are only available from 1999 to 2017, whereas the USGS statistics^21^ cover the period of 1990 to 2020 but they are measured in metal or concentrate content (*W*_*Metal ores in other units*_). Therefore, USGS statistics need to be converted with an empirical unit conversion factor (*P*_*Unit conversion factor*_) before being applied to estimate unavailable statistics reported by China, as shown in Eq. 6. Conversion factors are localised for each non-ferrous metal in each year from 2000 to 2017 by using USGS statistics divided by China statistics and then averaged after removing the highest value and the lowest value (i.e., trimmed mean). This factor could capture the general relationship between statistics from two separate sources, which can be used in other long time-series studies on resource management on a particular element in China.
    6 $${W}_{Metaloresingrossore}={W}_{Metaloresinotherunits}/{P}_{Unitconversionfactor}$$
  - **Non-metallic minerals:** The official China-specific information on non-metallic mineral domestic production is available between 1999 and 2017^20^, the rest of which could be estimated from USGS statistics (1990–2020)^21^. Also, two differences in reporting standards are observed resulting from the material coverages and reporting units. China statistics contain eighty-eight materials in mineral ores, whereas the USGS only includes twenty in the concentrate unit. Therefore, a conversion factor is developed in this estimation, as shown in Eq. 7. This conversion factor is applied to the total amount of non-metallic mineral production, which is assumed to have been constant from 1990 to 1999 at 11.38% (1999) and 12.56% (2017) from 2017 to 2020.
    7 $${W}_{Mineralsingrossore}={W}_{Mineralsinotherunits}/{P}_{Conversionfactor}$$
  - **Coal in fossil energy materials:** Coal, mined in China, includes raw coal, peat, stone coal, and oil shale. Except for raw coal, statistics for the rest are only available from 1999 to 2017^20^. The unavailable data (*W*_*Other coals*_) is estimated using Eq. 8 under the assumption that the structure of the coal supply in China barely changes. That is, the proportion (*P*_*Supply fraction*_) of peat, stone coal, and oil shale in raw coal production (*W*_*Raw coal*_) remains constant, so the 1999 proportion is applied to all years before that (earlier years of 1990–1998), while the 2017 proportion is used to the recent years between 2018 and 2020. For example, *P*_*Supply fraction*_ for oil shale production was assumed to be 0.014% during 1990–1999, calculated by dividing raw coal production (1,250,000) by oil shale production (179) in 1999. *P*_*Supply fraction*_ in the earlier and the recent years are 0.007% and 0.001% for peat, 0.203% and 0.031% for stone coal, and 0.014% and 0.067% for oil shale.
  8 $${W}_{Othercoals}={W}_{Rawcoal}/{P}_{Supplyfraction}$$
- **The output of processed materials by release**
  - **Materials released into the air:** In China, thirteen materials are released into the air, as shown in Fig. 1. The emission of sulphur dioxide (SO_2_) is reported in China environmental statistical yearbooks^22,23^, while the rest is specified in the EDGAR^24^. However, in EDGAR, statistics for recent years have not yet been updated, which are estimated with the value in the most recent year in our database. For example, nitrous oxide (NOx) records are only available for the years prior to 2016, with 26,365 thousand tonnes in 2015 and 26,837 in 2014. As a result of the observed decreasing trend in NOx emissions, NOx emission data for 2016–2020 is estimated to be 26,000 thousand tonnes. This estimate may be subjective due to constraints, but it would be aligned with European statistics, allowing for international comparisons. Data can be updated after the EDGAR statistics have been updated.
  - **Materials released into the water:** Ten principal materials have been found in China wastewater (both industrial and municipal) that are nitrogen (N), phosphorus (P), organic pollutants of petroleum, volatile phenol and cyanide, heavy metals of mercury (Hg), lead (Pb), cadmium (C·d), and the hexavalent chromium (Cr^6+^), and arsenic (As). Many statistics^22,23^ have been of poor quality (e.g., inconsistent material coverages between years). Given that the statistics of pollutants in industrial wastewater cover more periods and contain fewer abnormal observations, the total material emissions can be approximated from those of industrial wastewater. Equations 9 and 10 show the estimation processes. The materials in industrial wastewater (*W*_*Industrial materials*_) are first identified using material mass concentration (*P*_*Concentration*_) and the weight of industrial wastewater (*W*_*Industrial wastewater*_), and then the materials in total wastewater (*W*_*Total materials*_) are identified using the proportion (*P*_*Contribution*_) of materials in industrial wastewaters (*W*_*Industrial materials*_) to the total. The assumption is that *P*_*Concentration*_ and *P*_*Contribution*_ change gradually between years, which enables to use linear interpolation method to estimate unavailable parameters. Consider cyanide: its *P*_*Concentration*_ was 23.61 (1‰ ppm) in 2005 and 37.31 in 2002, which was assumed to be 28.18 in 2004 and 32.74 in 2003. *P*_*Concentration*_ was assumed to be 100% throughout the years for cyanide because all cyanide emissions in China are driven by industrial wastewater discharges. Later, the total material emissions can be derived by dividing the industrial wastewater mass by *P*_*Concentration*_.
    9 $${W}_{Industrialmaterials}={W}_{Industrialwastewater}\times {P}_{Concentration}$$
    10 $${W}_{Totalmaterials}={W}_{Industrialmaterials}\,/\,{P}_{Contribution}$$
  - **Materials released to the** **land:** This is zero because uncontrolled landfills are illegal in China.
  - **Materials dissipated by organic fertiliser use:** In China, manure is the primary organic fertiliser, which is excreted by pigs, dairy cows, calves, sheep, horses, asses, mules, camels, chickens, and other animals. As shown in Eq. 11, the manure production (*W*_*Manure*_) is estimated through the amounts of raised livestock (*Q*_*Livestock*_, heads), the weight of daily manure production (*P*_*Manure production*_, kilograms per head per day), the number of days they are raised (*P*_*Feeding period*_, in days per year), and the moisture content of their manure (*P*_*Dry matter*_, %) for each type of animal. These parameters are region-specific, which have been localised by Chinese scholars^25–27^ and listed in Table 2.
    11 $${W}_{Manure}={Q}_{Livestock}\times {P}_{Manureproduction}\times {P}_{Feedingperiod}\times {P}_{Drymatter}$$

**Table 2:** Localised parameters for animal manure production.

Animals	Daily production	Feeding period^25^	Dry matter^4^
Pigs (Slaughtered)	5.3^25^	199	0.071
Dairy cows (Year-end stocks)	53.15^25^	365	0.085
Calves (Slaughtered)	21.1^25^	365	0.05
Other bovine (Year-end stocks)	27.67^25^	365	0.085
Sheep (Year-end stocks)	2.25^26^	365	0.07
Horses (Year-end stocks)	16.16^25^	365	0.07
Asses (Year-end stocks)	13.9^26^	365	0.07
Mules (Year-end stocks)	13.9^26^	365	0.07
Camels (Year-end stocks)	13.7^26^	365	0.07
Broilers chickens (Slaughtered)	0.1^25^	55	0.15
Laying hens (Year-end stocks)	0.15^25^	365	0.15
Other poultry (Slaughtered)	0.107^25^	210	0.15
Rabbit and hares (Slaughtered)	0.37^26^	90	0.15
  - **Materials dissipated by mineral fertiliser use:** The mineral fertilisers used in China are four types, i.e., nitrogen (N), phosphorus (P), potash (K), and compound. Their usage (*W*_*Fertiliser usage*_) is measured in nutrient mass (*W*_*Nutrient materials*_), which needs to be converted into the gross mass by dividing their nutrient content (*P*_*Nutrient content*_). Equation 12 shows the estimation. This parameter of *P*_*Nutrient content*_ is localised by the Ministry of Agriculture and Rural Affairs of China^28^ as 29%, 22%, 35%, and 44% for N- bearing, P- bearing, K-bearing, and compound fertilisers, respectively.
    12 $${W}_{Fertiliserusage}={W}_{Nutrientmaterials}/{P}_{Nutrientcontent}$$
  - **Materials dissipated by sewage sludge:** Sewage sludge is the residue generated by municipal wastewater treatment. As demonstrated in Eq. 13, its dissipative use (*W*_*ss, dissipation*_) is the untreated amount of production (*W*_*ss, production*_), represented by the parameter of *P*_*ss, dissipation rate*_. Sewage sludge production (*W*_*ss, production*_) statistics are only available for the years 2006–2020^29^, and data for the remaining years can be estimated using Eq. 14 and Eq. 15. In Eq. 14, *P*_*ss, production rate*_ represents the relationship between sewage sludge production (*W*_*ss, production*_, 2006–2020) and wastewater treatment (*W*_*ww, treatment*_, 2002–2020), and in Eq. 15, *P*_*ww, treatment efficiency*_ represents the relationship between the quantity of treated wastewater (*W*_*ww, treatment*_, 2002–2020) and the treatment capacity (*W*_*ww, treatment capacity*_, 1990–2020). In this estimation, three assumptions are made. The first is to estimate *W*_*ww, treatment*_, *P*_*ww, treatment efficiency*_ is assumed to be unchanged at 63% during 1990–2001, given it has been increasing from 63% in 2002 to ~80% in recent years. The second is that, in order to estimate *W*_*ss, production*_, *P*_*ss, production rate*_ is assumed to be unchanged at 3.5 between 1990 and 2005, suggesting 3.5 tonnes of sewage sludge are generated by processing 10,000 cubic metres of wastewater. This assumption is determined by that *P*_*ss, production rate*_ is approximately 3.5 during 2006–2010 while declines sharply and stabilises at around two during 2011–2020. The last is, to estimate the *W*_*ss,dissipation*_, *P*_*ss,dissipation rate*_ is assumed to be 5% between 1990 and 2005, given it has been around 5% during 2006–2020.
    13 $${W}_{ss,dissipation}={W}_{ss,production}\times {P}_{ss,dissipationrate}$$
    14 $${W}_{ss,production}={W}_{ww,treatment}\times {P}_{ss,productionrate}$$
    15 $${W}_{ww,treatment}={W}_{ww,treatmentcapacity}\times {P}_{ww,treatmentefficiency}$$
  - **Materials dissipated by composting:** Composting is a natural process that uses microbes to turn organic materials into other products, which are then used for fertilising and entering the environment. In China, composting has been used to treat two materials: feces and municipal waste, whose quantities (*W*_*Composting*_) were only available from 2003 to 2010^29^. The unavailable data can be estimated using Eq. 16. The dry weight of materials treated by composting (*W*_*Composting*_) is proportionally related to the fresh weight of all treated materials (*W*_*Total*_), the proportion treated by composting (*P*_*Composting rate*_), and the dry content (*P*_*Dry matter*_). Considering that China’s composting capacity has been declining since 2001 due to the implementation of waste incineration power generation technologies^30^, *P*_*composting rate*_ is assumed to be the same as it was in 2003 (9.5%) between 1990 and 2002, and 1.5% in 2010 between 2011 and 2020. The parameter of *P*_*Dry matter*_ is 50%^4^.
    16 $${W}_{Composting}={W}_{Total}\times {P}_{Compostingrate}\times {P}_{Drymatter}$$
  - **The input and output by cross-border trade**. Statistics of imports and exports have been gathered since 1962 and stored in the UN Comtrade database^31^. However, the data quality issue of outliers, and missing values, especially in weight, is reportedly identified. In our previous work, we addressed these issues, and an improved database^32^ is provided. Details about our estimation methods can be found in publications^33–35^. As UN Comtrade lists 5,039 different commodity types (in 6-digit HS0 commodity code), yet only 18 material types are specified in the China EW-MFA, UN Comtrade statistics need to be aligned to the China EW-MFA framework. Therefore, we compared each commodity and each material type between them and established a correspondence table to map UN Comtrade commodity types onto our EW-MFA material types. For example, non-ferrous metal materials of China EW-MFA include commodities, such as copper ores and concentrates (260300 HS0 code), silver powder (710610), manganese, articles thereof, and waste or scrap (811100), etc., whereas biomass residues include cereal straw and husks (121300), lucerne meal and pellets (121410), and other fodder and forage products (121410). This correspondence table between HS0 and EW-MFA classification for imports and exports is provided in Supplementary File 1.
- **The input of balancing items**
  - **O**_**2**_ **required for combustion:** In ***BI***, requirements for materials can be abstracted as equalling exogenous demands minus intrinsic supplies (Eq. 17). Three parts (two demands and one supply) are considered for O_2_ requirements by the combustion process: (1) demanding exogenous oxygen to oxidise elements (e.g., carbon, sulphur, nitrogen, etc., except for hydrogen) released into the air, (2) demanding exogenous oxygen to oxidise the hydrogen embedded in fossil energy materials, and (3) providing intrinsic oxygen embedded in fossil energy materials. The first part can be estimated via Eq. 18 by multiplying air emissions (*W*_*Emissions*_) of CO_2_, N_2_O, NO_x_, CO, and SO_2_ by their oxygen content (*P*_*Oxygen content*_). For the second (Eq. 19), the oxygen demand is estimated based on the principle of mass balance by converting the hydrogen amount of domestically utilised fossil energy materials (*W*_*Fossil fuel materials*_ × *P*_*Hydrogen content*_) via molar mass conversion factor (*P*_*Mass conversion factor*_). *P*_*Mass conversion factor*_ equals 7.92, derived by the molar mass of one oxygen (16 g/mol) divided by that of two hydrogen atoms (2 × 1.01 g/mol). The last is the intrinsic supplies from fossil fuel materials, which is identified via Eq. 20 by multiplying the domestically utilised amount of fossil fuel materials (*W*_*Fossil fuel materials*_) by their oxygen content (*P*_*Oxygen content*_). The parameters in this estimation are presented in Table 3. As a footnote here, the domestically utilised amount is referred to as the domestic material consumption (***DMC***), which equals domestic extraction (***DE***) plus imports (***IM***) and minus exports (***EX***).
    17 $${W}_{Requirements}={W}_{Demands}-{W}_{Supplies}$$
    18 $${W}_{Demands}={W}_{Emissions}\times {P}_{Oxygencontent}$$
    19 $${W}_{Demands}={W}_{Fossilfuelmaterials}\times {P}_{Hydrogencontent}\times {P}_{Massconversionfactor}$$
    20 $${W}_{Supplies}={W}_{Fossilfuelmaterials}\times {P}_{Oxygencontent}$$

**Table 3:** Parameters related to combustion processes^4^.

Materials		Content			
		Oxygen	Hydrogen	Others	Moisture
Air emissions	CO_2_	73%	—	27%	—
	N_2_O	36%	—	64%	—
	NO_x_	70%	—	30%	—
	CO	57%	—	43%	—
	SO_2_	50%	—	50%	—
Fossil fuel materials	Wood fuel and others	35%	5%	45%	15%
	Raw coal	10%	3%	37%	50%
	Stone coal	4%	5%	76%	15%
	Oil shale and tar sands	0%	2%	91%	7%
	Peat	25%	7%	34%	35%
	Crude oil, condensate and natural gas liquids	0%	12%	87%	1%
	Natural gas	0%	25%	75%	0%
  - **O**_**2**_ **required for respiration:** O_2_ is required by the metabolic activities of living organisms, the majority of which are humans and livestock. Bacteria are another sort of organism, which are not included in this estimation because their O_2_ requirements are too small to be quantified. The respiration-required O_2_ is related to the total quantity (*Q*_*Organisms*_) and their respiration activity by organism types, as shown in Eq. 21. The respiration activity is represented by the respiration requirement coefficient (*P*_*Respiration requirement* coefficient_), which is the average quantity of O_2_ that each organism utilises to maintain the metabolic activity, as listed in Table 4.
    21 $${W}_{Demands}={Q}_{Organisms}\times {P}_{Respirationrequirementcoefficient}$$

**Table 4:** Parameters related to respiration processes^4^.

Organisms		Requirement coefficients	Emission coefficients	
		O_2_	CO_2_	Water vapour
		Unit: tonnes per head per year		
Humans		0.25	0.3	0.35
Cultivated livestock	Cattle	2.45	2.92	3.38
	Sheep	0.2	0.24	0.27
	Horses	1.84	2.19	2.53
	Pigs	0.25	0.3	0.35
	Poultry	0.01	0.01	0.01
  - **Water required for the** **domestic** **production of exported beverages:** The exported beverages are produced domestically using domestically extracted materials, especially a large amount of water. The weight of water is considered in the output by cross-border trade but is not included in the domestic extraction input. The resulted imbalance can be identified by specifying the water weight in beverages, i.e., multiplying the traded beverage weight (*W*_*Materials*_) by a parameter of the water content (*P*_*Water content*_), as given in Eq. 22. Fruit and vegetable juices (2009 in HS0 code) and beverages (code 22) are covered in the improved UN Comtrade database^32^, with *P*_*Water content*_ of 85% for the first and 90% for the latter^4^.
  22 $${W}_{Water}={W}_{Materials}\times {P}_{Watercontent}$$
- **The output of balancing items**.
  - **Water vapour from combustion:** Water vapour emissions by domestically combusting fossil fuel materials are contributed by two paths. The direct evaporation of embedded water is the first path (Eq. 23), which can be derived by multiplying the ***DMC*** of fossil fuel materials by their moisture content (*P*_*Moisture content*_). The *P*_*Moisture content*_ for each type of fossil fuel material is listed in Table 3. The other is the generation of water vapour during hydrogen oxidation, which can be calculated by converting the oxidised weight of hydrogen to the water weight using the molar mass conversion factor (*P*_*Mass conversion factor*_), as given in Eq. 24. *P*_*Mass conversion factor*_ equals 8.92 by dividing the molar mass of water (18.02 g/mol) by that of two hydrogen atoms (2 × 1.01 g/mol).
    23 $${W}_{Water}={W}_{Fossilfuelmaterials}\times {P}_{Moisturecontent}$$
    24 $${W}_{Water}={W}_{Fossilfuelmaterials}\times {P}_{Hydrogencontent}\times {P}_{Massconversionfactor}$$
  - **Water vapour and CO**_**2**_ **from respiration:** Respiration activities of organisms will produce water vapour and CO_2_, whose estimation is similar to that of O_2_ requirements. As shown in Eq. 25, the respiration-caused gas emissions are related to the number of organisms (*Q*_*Organisms*_) and the respiration activity by organism types. The latter is represented by the parameter of respiration emission coefficient (*P*_*Respiration emission* coefficient_), which is specified in Table 4 for water vapour and CO_2_ for each type of organism.
    25 $${W}_{Emissions}={Q}_{Organisms}\times {P}_{Respirationemissioncoefficient}$$
  - **Water from imported beverages:** The estimation approach is the same as water by the domestic production of exported beverages, as described in Eq. 16.
  - **Water in biomass products:** Usually, the input of biomass products by domestic extraction^16^ has been measured in fresh weight, but their corresponding output^29^ by sewage sludge, composting, etc., are in dry weight, leading to an imbalance in water weight. The water weight in biomass products is calculated by multiplying their domestic extraction amount in fresh weight (*W*_*Biomass*_) by a parameter of moisture content at harvest (*P*_*Moisture content*_), as shown in Eq. 26. The values of *P*_*Moisture content*_ by biomass products are presented in Table 5.

**Table 5:** The moisture content at harvest for each biomass product^4^.

Biomass product	The moisture content at harvest
Cereals	14%
Sugar crops	77%
Pulses	10%
Nuts	5%
Oil-bearing crops	22%
Vegetables	93%
Fruits	78%
Fibres	11%
Other crops	8%

26 $${W}_{Water}={W}_{Biomass}\times {P}_{Moisturecontent}$$

### Material flow quantification

The above attempts have quantified material inputs and outputs by flows and presented a detailed profile of material utilisation for each material in China’s economy. In order to depict the economy in a more general way, EW-MFA indicators are assessed by aggregating flows by materials or periods as below.

- **Domestic extraction (*DE*)**: is referred to as natural materials that are extracted from the domestic environment and are used in the domestic economy, i.e., the total input of natural materials by extraction.
- **Domestic processed output (*DPO*)**: is referred to as materials that are released to the domestic environment after being processed in the domestic economy, i.e., the total output of processed materials by release.
- **Import (*IM*)**: is referred to as all goods (in the form of raw materials, semi-finished materials, and final products) that originated from other economies and are further used in the domestic economy. It is calculated as the sum of all imported goods.
- **Export (*EX*)**: is referred to as all goods that originated from the domestic economy and are transported to other economies to be used. It is calculated as the sum of all exported goods.
- **Domestic material input (*DMI*)**: is referred to as materials that originated from the domestic environment by extraction and other economies and are available (to be used or to be stored) for the domestic economy. It is calculated as the sum of DE plus IM, as shown in Eq. 27.
  27 $$DMI=DE+IM$$
- **Domestic material consumption (*DMC*):** is referred to as materials that are directly used in the **domestic** economy after parts of them are exported to other economies. It is calculated as the difference between ***DMI*** and ***EX***.
- **Physical trade balance (*PTB*):** is referred to as a surplus or deficit of materials for the domestic economy. It is **calculated** as the difference between ***IM*** and ***EX***.
- **Net additions to stock (*NAS*):** is referred to as materials that remain in the domestic economy. It is calculated by taking ***BI*** items into account, as shown in Eq. 28.

28 $$NAS=DMC+B{I}_{in}-DPO-B{I}_{out}$$

## Data Records

A total of seven data files are accessed publicly at figshare repository^36^ and at our online website(https://www.macycle.org/china-ewmfa/). Five Excel files in .xlsx format provide detailed information on data compilation, including original statistics, data sources, and estimation methods (using formulas). Being interoperable and reusable, the ready-to-use China EW-MFA data are prepared in two files (***EWMFA_Flows.csv*** and ***EWMFA_Flows.json***). Data records in each file are introduced in brief as follows.

**Fig. 2:**
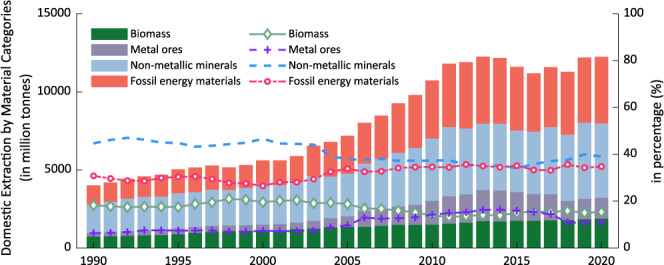
Domestic extraction of natural materials by China’s economy during 1990–2020.
  More specifically, Fig. 3 presents the domestic extraction of each resource from 1990 to 2020. Among biomass materials extracted, crops accounted for the majority with an average of 1,300 million tonnes, followed by residues (165), wild products (45), and wood (3.5). Details of, for example, cereals in crops, grazed biomass in residues, and timber in wood, can be found in Supplementary File 2. The extraction of iron ores grew dramatically from 1990 (168 million tonnes) to 2013 (1522) but decreased recently to 866 in 2020, with a ten-year low of 763 in 2018. Furthermore, the extraction of non-ferrous metal ores has been no more than 600 million tonnes, with copper (Cu), gold (Au), and molybdenum (Mo) accounting for the majority. In fossil energy materials, over 95% are occupied by coal, with crude oil and natural gas following. There is a rising trend in the extraction of natural gas. The annual coal extraction increased from 1,082 million tonnes in 1990 to 3,905 in 2020, with a peak at 3,978 in 2013 and a slight drop to 3,412 in 2016. Differently, crude oil extraction has been stabilising in the range of 130–200 million tonnes per year.

- ***Domestic Extraction.xlsx***: This file contains original statistics as well as data sources and estimated data related to the quantification of ***DE***. from 1990 to 2020. In addition, the compilation tools for ***DE*** estimation as described above are implemented in the file using formulas only. As shown in Fig. 2, China extracted 4,178 million tonnes of natural materials in 1990, but this figure is three times larger (12,462) in 2020. The total ***DE*** increased rapidly from 1990 to 2010 and then remained flat from 2011 to 2014, with a slight decrease after 2015. In addition, the percentage of each material changed slightly. The extraction of non-metallic minerals has accounted for the majority, followed by fossil energy materials. However, the gap between them has shrunk, which was almost the same during 2012–2014. This percentage of biomass has been falling, with 20% in 2000 but 15% in 2020. With regard to metal ores, their ***DE*** percentage increased slightly from 2000 (7%) to 2015 (16%) but then fell to 10% in 2020.

**Fig. 3:**
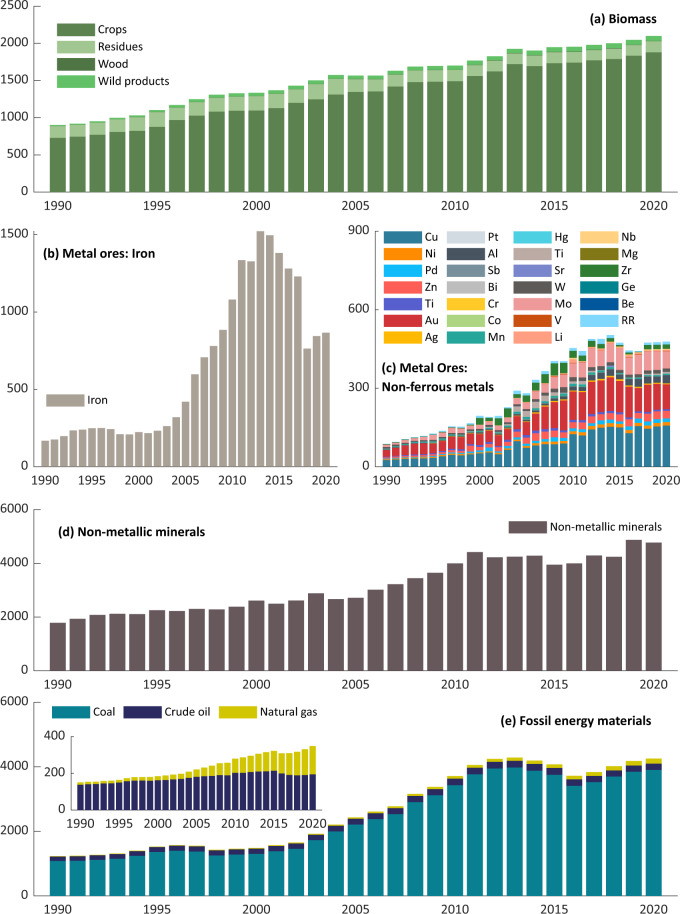
Domestic extraction of each material by China’s economy during 1990–2020, by biomass (**a**), metal ores of iron (**b**) and non-ferrous metals (**c**), non-metallic minerals (**d**), and fossil energy materials (**e**). Values are measured in a million tonnes.
- ***Domestic Processed Output.xlsx***: This file contains original statistics as well as data sources and estimated data related to the quantification of ***DPO*** from 1990 to 2020. In addition, the compilation tools for data estimation as described above are implemented in the file using formulas only. As indicated in Fig. 4(a), the number of materials released from China’s economy to the environment has been increasing from 2,869 in 1990 to 12,710 million tonnes in 2020, which is six-fold growth at a rate of 330 million tonnes per year. Between 2000 and 2013, there was a significant increase in material output. The figure has risen from 4,070 in 2000 to 11,510 in 2013, with an annual growth rate of 572 million tonnes. It is worth emphasising that the CO_2_ emissions to the air contribute to the majority (greater than 90%) of the ***DPO****,* with 92.4% in 1990 and 98% in 2020, as shown in Fig. 4(b). Other emissions to air (Fig. 4(c)) also show an increasing trend, primarily contributed by CO and CH_4_. As shown in Fig. 4(d), the ***DPO*** to water is found to be decreasing from 9.5 in 1991 to 1.2 million tonnes in 2019, suggesting an improved water quality in China. N is the primary material in ***DPO*** to water, which is sourced from fertiliser applications and discharges from livestock, domestic and industrial sources^37^. Sharp decreases in N emissions are observed from 4.61 million tonnes in 2015 to 1.24 in 2016, which can be ascribed to China’s 13^th^ Five Year Plan’s efforts to upgrade urban sewage systems, increase wastewater treatment rates, and reduce fertiliser usage^38^. However, a rise is observed in 2020, which may be attributed to rising municipal wastewater volumes during the pandemic period and the corresponding increasing emission of N^39^. The rest materials released into the water are mostly released by industrial wastewater, whose emission has been declining from 0.105 in 1990 to 0.004 (3%) million tonnes in 2020. The emissions by dissipative use (Fig. 4(e)) are primarily caused by the utilisation of organic (average at 215 million tonnes) and mineral fertilisers (150), followed by compos (2.2), pesticides (1.4), and sewage sludge (0.2). The ***DPO*** to land and through dissipative loss are zero in China.

**Fig. 4:**
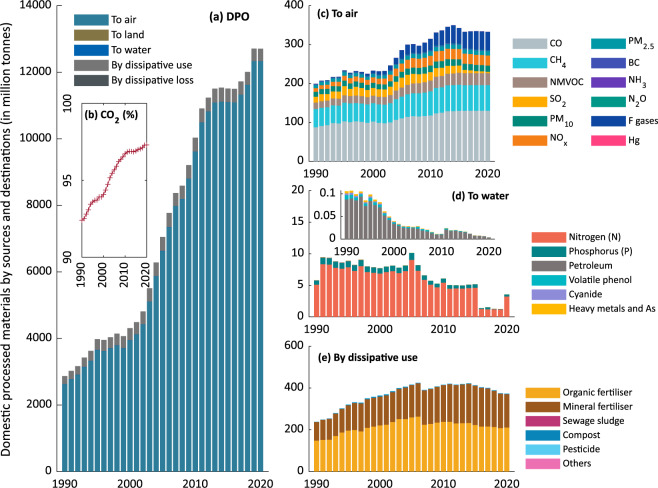
Output of domestic processed material by sources and destinations during 1990–2020. Values are measured in a million tonnes. (**a**) is the total amount; (**b**) is the percentage that output of CO_2_ accounts for the total ***DPO***; the number of each material to air (**c**), to water (**d**) and by dissipative use (**e**).
- ***Balancing Items.xlsx:*** This file contains original statistics as well as data sources and estimated data related to the quantification of ***BI*** from 1990 to 2020. In addition, the compilation tools for data estimation as described above are implemented in the file using formulas only. The ***BI*** for both input and output is dominantly contributed by the combustion process, followed by the respiration process, as shown in Fig. 5. To note, Fig. 5(a) shows abrupt increases in the amount of water needed to domestically produce exported beverages, which are ascribed to significant exports to China’s special administrative region of Hong Kong in 2000 (43.2 million tonnes), 2001 (43.4), 2003 (36.7), 2011 (14.4) and 2012 (68.7). In Fig. 5(b). the significant increase in the imported beverages’ water was the result of large imports from Japan in 2012 while the United States and France during 2016–2018.

**Fig. 5:**
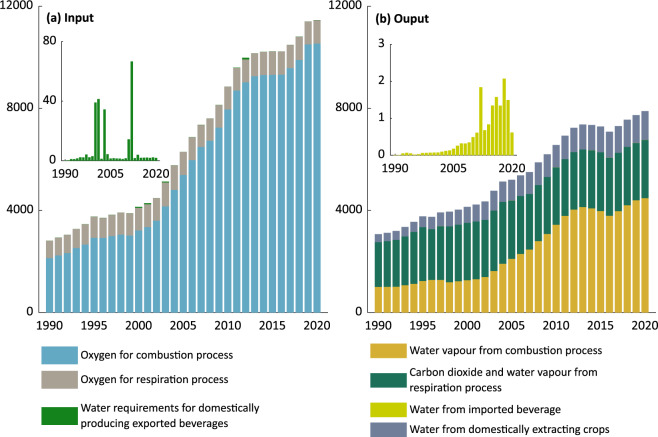
***BI*** by input (**a**) and output (**b**). Values are measured in a million tonnes.
- ***Imports and Exports.xlsx:*** This file contains data records of all imported and exported goods, by EW-MFA classifications of materials and by flows of ***IM*** and ***EX***. As shown in Fig. 6(a), from 1990 to 2019, China’s economy imported a rising number of materials, which had a drop in 2020. The figure for 2020 is 1,619 thousand tonnes, which is roughly half of the total for 2019 (2,779). The imported materials are mostly metal ores and fossil energy materials, followed by biomass and non-metallic minerals. Regarding exported materials in Fig. 6(b), the total amount has continuously increased from 10 in 1990 to 864 thousand tonnes in 2020 (more than eighty times).

**Fig. 6:**
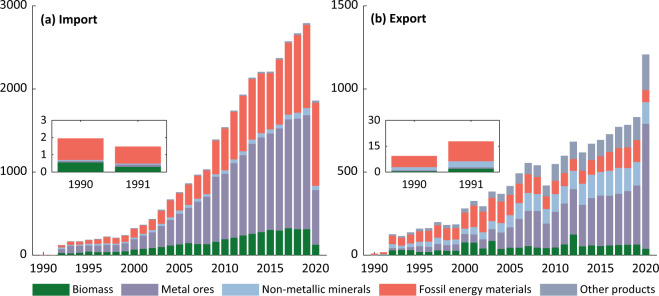
The ***IM*** (**a**) and ***EX*** (**b**) by materials. Values are measured in a thousand tonnes.
- ***China EW-MFA Accounts (1990***–***2020).xlsx:*** It contains flows and derived indicators from 1990 to 2020 for China’s economy and for each material category, which is the result calculated from the above statistics. Indicators are ***DE***, ***IM***, ***EX***, ***DMI***, ***DMC***, ***NAS***, and ***PTB***, for **China’s** economy, biomass, metal ores, non-metallic minerals, and fossil energy materials respectively, while ***BI***, and ***DPO*** for China’s economy. Figure 7(a) shows the number of materials that China’s economy inputs, uses, and stores each year, and the percentage of ***IM*** in ***DMI***, which represents the import dependency. Both ***DMI*** and ***DMC*** have experienced remarkable growth, with no significant difference between them. This indicates that most input materials are utilised by China’s economy, and China has been rapidly developing. Moreover, this development has been intensively utilising domestic resources, given that ~90% of ***DMI*** is contributed by ***DE***. However, the percentage of ***IM*** in ***DMI*** has been continuously increasing from 2.5% in 1992 to 18.3% in 2019, indicating that China’s economy is becoming more reliant on imported resources. This greater dependence is also observed with a gradual increase of ***PTB*** in Fig. 7(b), suggesting an increasing difference between imports and exports.

**Fig. 7:**
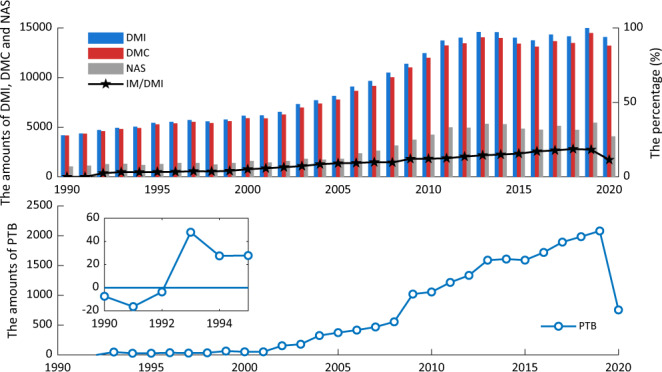
The EW-MFA indicators for China’s economy, with ***DMI***, ***DMC***, ***NAS****,* and ***IM/DMI*** in (**a**) and ***PTB*** in (**b**). Values are measured in a million tonnes.
- **EWMFA_Flows.csv and EWMFA_Flows.json**: These two files contain the whole dataset (4,620 data cells) in an interoperable **format**, which can be directly applied in studies.

## Technical Validation

### Data validation

Regarding our study, this database is designed to provide officially verified data that are related to the material flows in China by collecting and compilating China-specific statistics. Therefore, the data quality of this database is ensured by the official statistics’ data quality. Our data validation should be established to verify that any data values are consistent with those officially published statistics. This is verified by manually reviewing any data values repeatedly using a developed data quick-check tool. This tool connects every data cell to its source (in any format), making it easy and convenient for a manual double-check of data consistency to improve the reliability of this study. This tool was developed with the Office Visual Basic for Applications (VBA) and is built into the data collection files. The instructions and source codes for this tool can be found in Supplementary File 3 and Supplementary File 4. To note, this tool can be applied to any data collection procedure. Additionally, by reviewing any graphical presentations of the data (Figs. 2–7), extreme values, outliers, and missing values are detected and verified, and the results show there is a good continuity and consistency in time and categories. That is to say that our dataset may be applicable for supplementing the existing data, and thus could provide the most long-term and up-to-date metabolic information about China’s economy.

### Uncertainties, limitations, and future work

In this study, uncertainties stem from artificial errors during statistic collection and subjective parameter selection during data compilation.

- The major uncertainty of this constructed database is caused by artificial mistakes (e.g., vision errors) during statistic collection procedures. Trying to reduce this, we repeatedly review the data values of this dataset and compare them with the officially-published statistics. So far, this statistic collection procedure still must be done manually, because original statistical publications are unavailable for direct use. The reasons are (1) most original statistical publications are stored in only human-understandable data format, and (2) these formats and also statistical criteria (e.g., units, material coverages) have been continuously changing. As a result, before statistics are applied, these publications must be compared and converted, both of which must be done manually. So far, this study has integrated compilation methods with a pre-defined data format, allowing the material flows to be determined correspondingly by re-organizing the collected statistics into each data cell. It works like a calculator, with statistics in and material flow results out, which to a large extent has reduced uncertainties in the flow quantification. For future studies, the focus could be put on converting original statistical publications into a data format that a machine can directly understand and process, using technologies, such as pattern recognition and natural language processing. These efforts could reduce human-related uncertainties to a larger extent.
- In data estimation, parameter uncertainties always exist since parameters are rarely perfectly accurate. In this study, trying to mirror the reality as closely as possible, we used most of the parameters that had been localised as China-specific by previous studies. For example, *P*_*Nutrient content*_ for China-used mineral fertilisers has been identified by the China statistical department. However, some parameters have not been localised, for example, the moisture content of fossil fuel materials in China. These parameters were adopted as indicated by the Eurostat^4^, which could allow international comparisons but may lead to differences between the estimated results and the facts. In addition, uncertainties may inevitably stem from our subjective parameter assumptions, that parameters are assumed to be static or changing linearly during a period. These assumptions may cause under-or overestimation of flows and related indicators. For example, the yield of vegetable production (*P*_*Yield*_) is assumed to be unchanged from 1990 to 1994, which may result in small biases in estimations of ***DE***, ***BI*** (Output of water in biomass products), and other indicators. However, minor uncertainties are likely to arise because only a small percentage of flows are estimated based on assumptions.

## Supplementary information

Supplementary File 2

Supplementary File 4

Supplementary File 3

Supplementary File 1

## Data Availability

All the results were generated using the basic formulas in Excel (version 2204). Along with results, all formulas are built into our Data Record files (*Domestic Extraction.xlsx*, *Domestic Processed Output.xlsx, Imports and Exports.xlsx, Balancing Items.xlsx*, and *China EW-MFA Accounts (1990*–*2020).xlsx*) at figshare repository^36^. The tool for checking data consistency is developed with the Office Visual Basic for Application (VBA), which can be built into any Excel file. The instructions and source codes are provided in Supplementary File 3 and Supplementary File 4. These codes are publicly available.
