# Supplementary material for: China economy-wide material flow account database from 1990 to 2020
Source: Sci Data. 2022 Aug 17;9:502. doi: 10.1038/s41597-022-01611-z (PMC9385661; doi:10.1038/s41597-022-01611-z)
Supplement: Supplementary file 1 — Supplementary File 2 [file 41597_2022_1611_MOESM1_ESM.docx]

**Supplementary figure**


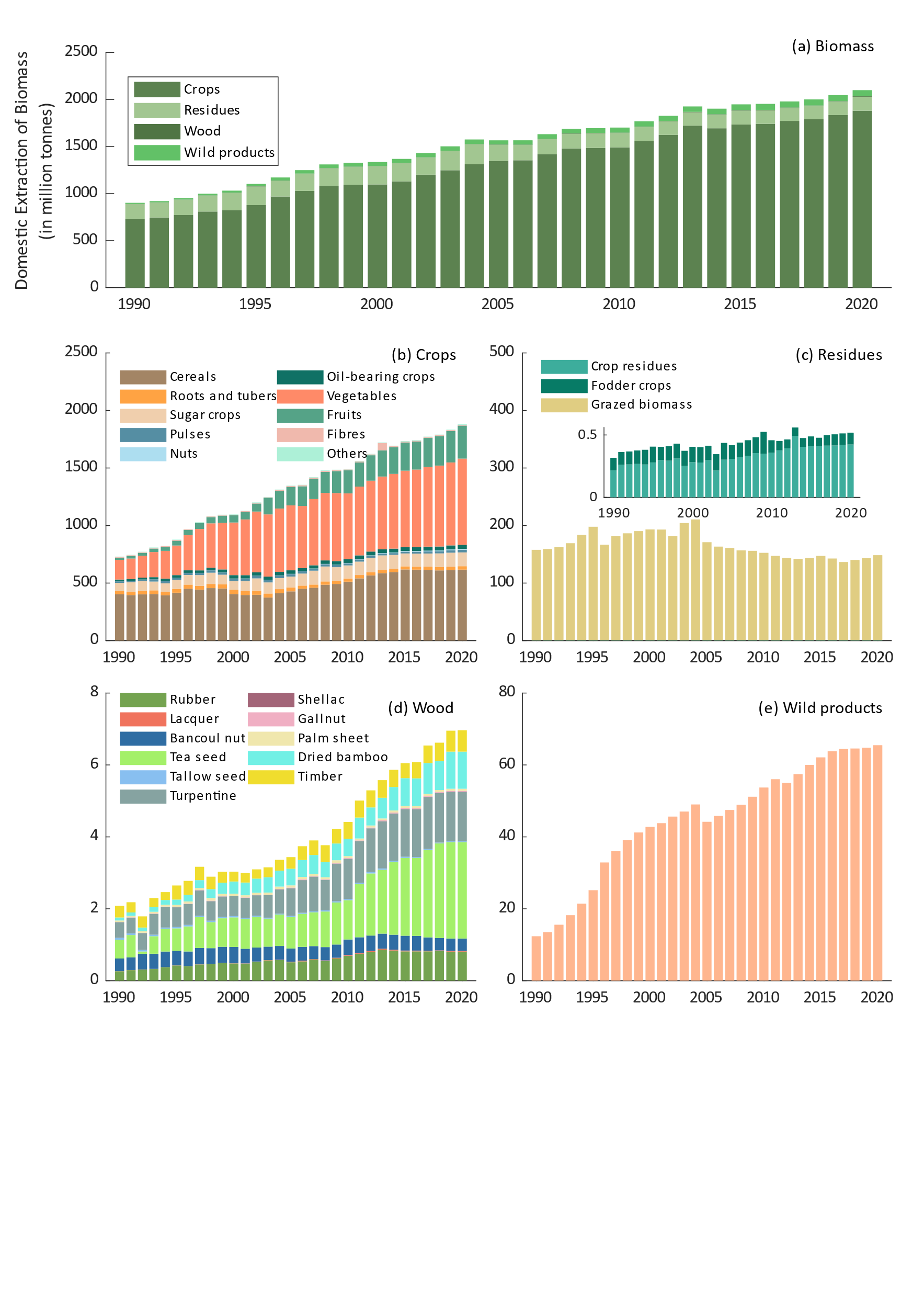


Figure 1 Domestic extraction of biomass materials into the China’s economy during 1990-2020, with the total amount of biomass (a), crops (b), residues (c), wood (d) and wild products (e). The values are measured in the unit of million tonnes.
