# Supplementary material for: China economy-wide material flow account database from 1990 to 2020
Source: Sci Data. 2022 Aug 17;9:502. doi: 10.1038/s41597-022-01611-z (PMC9385661; doi:10.1038/s41597-022-01611-z)
Supplement: Supplementary file 2 — Supplementary File 4 [file 41597_2022_1611_MOESM2_ESM.docx]

**The double-check tool: Source Code (with Visual Basic for Applications)**

1. Sub showFootnote_Click()
2. Dim footNoteSheet
3. Dim footNoteStr As String
4. Dim sourceStr As String
5. Dim isFootNote As Boolean
7. Set footNoteSheet = Sheets("Sources")
8. footNoteStr = ""
9. sourceStr = ""
10. isFootNote = False
12. If ActiveCell.Cells Like "*)" Then
13. footNoteStr = ActiveCell.Cells
14. isFootNote = True
15. Else
16. footNoteStr = Sheets("Statistics").Cells(ActiveCell.Cells.Row, ActiveCell.Cells.Column + 1)
17. If footNoteStr Like "*)" Then isFootNote = True
18. End If
20. If isFootNote Then
21. If footNoteStr = "e)" Then
22. isFootNote = False
23. MsgBox "By estimation"
24. Else
25. For i = 3 To 204
26. If footNoteSheet.Cells(i, 1) = footNoteStr Then
27. getSource ("Source API Url=" + Trim(footNoteSheet.Cells(i, 2)))
28. *%% Source API Url: personal setting.*
29. MsgBox "Downloading... " + footNoteSheet.Cells(i, 2)
30. End If
31. Next i
32. End If
33. Else
34. MsgBox "Error!!"
35. End If
36. End Sub
37. Sub getSource(objUrl As String)
38. Dim objRequest As Object
39. Dim blnAsync As Boolean
40. Dim strResponse As String
41. Set objRequest = CreateObject("MSXML2.ServerXMLHTTP")
43. blnAsync = True
44. With objRequest
45. .Open "GET", objUrl, blnAsync
46. .SetRequestHeader "Content-Type", "application/json"
47. .Send
48. While objRequest.ReadyState <> 4
49. DoEvents
50. Wend
51. strResponse = .ResponseText
52. End With
54. ActiveWorkbook.FollowHyperlink strResponse
55. End Sub
