# Supplementary material for: China economy-wide material flow account database from 1990 to 2020
Source: Sci Data. 2022 Aug 17;9:502. doi: 10.1038/s41597-022-01611-z (PMC9385661; doi:10.1038/s41597-022-01611-z)
Supplement: Supplementary file 3 [file 41597_2022_1611_MOESM3_ESM.docx]

**The double-check tool: User Guides**

Here, the procedures of data retrieval, source file storage, and double-checking will be presented. For more information, please email us.

**Step 1: Retrieve the original statistic file in any format.**

As shown in Figure 1, emissions of carbon dioxide (CO_2_) during 1970-2020 are reported in the EDGAR database (<https://edgar.jrc.ec.europa.eu/country_profile/CHN>), and these reported data (referred to as source files) were saved as a screenshot in png format and named as ‘EDGAR1970-2020_CO2.png’.


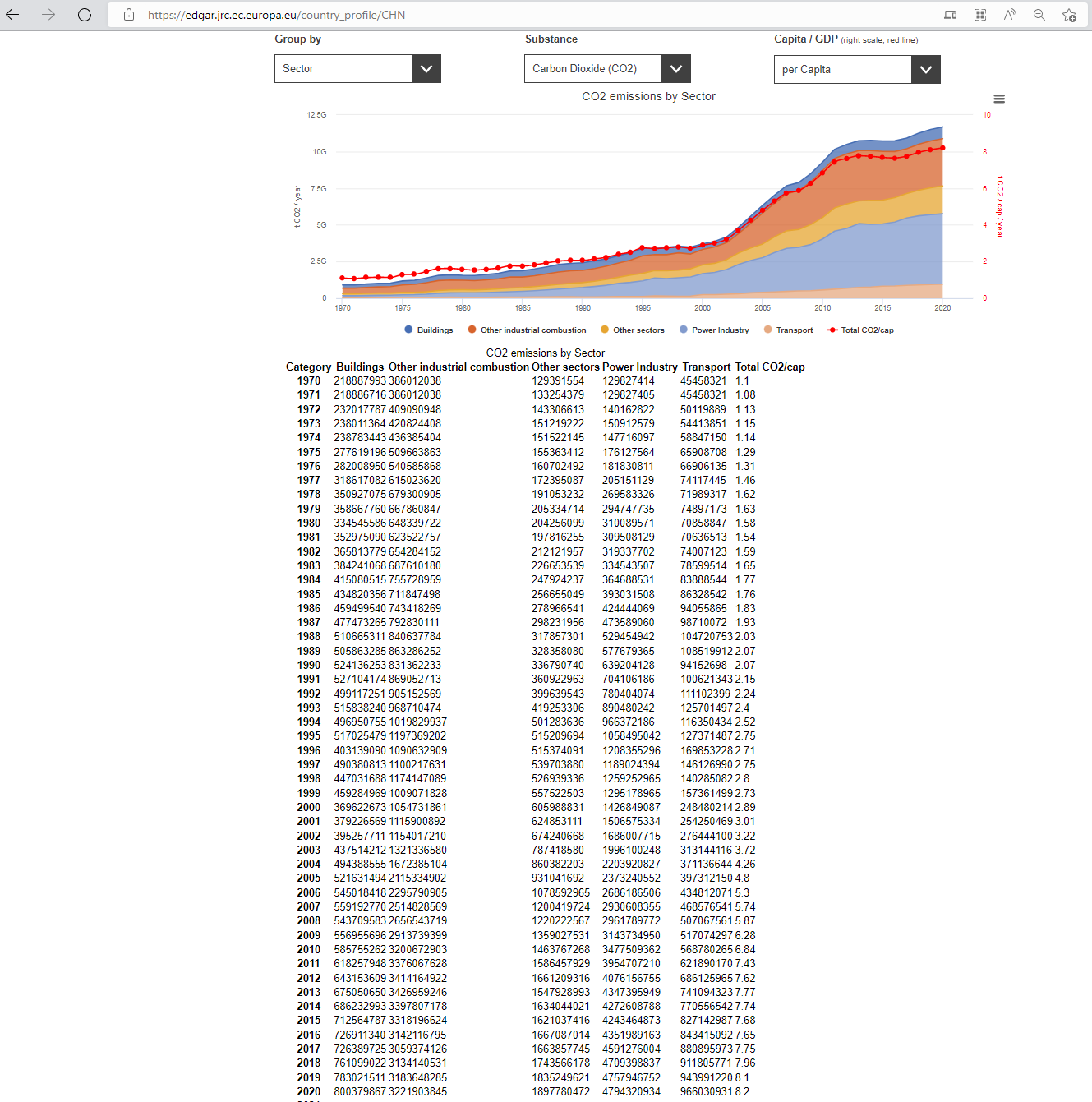


Figure 1 The file ‘EDGAR1970-2020_CO2.png’

**Step 2: Store these source files.**

Based on the Object Storage Service (https://www.aliyun.com/product/oss), these source files are uploaded by publications. For example, the air emissions from the EDGAR database are stored in .png format (Figure 2), while domestic extractions of minerals from China Land and Resources Statistical Yearbooks are stored in tables in .xlsx or .pdf format (Figure 3).


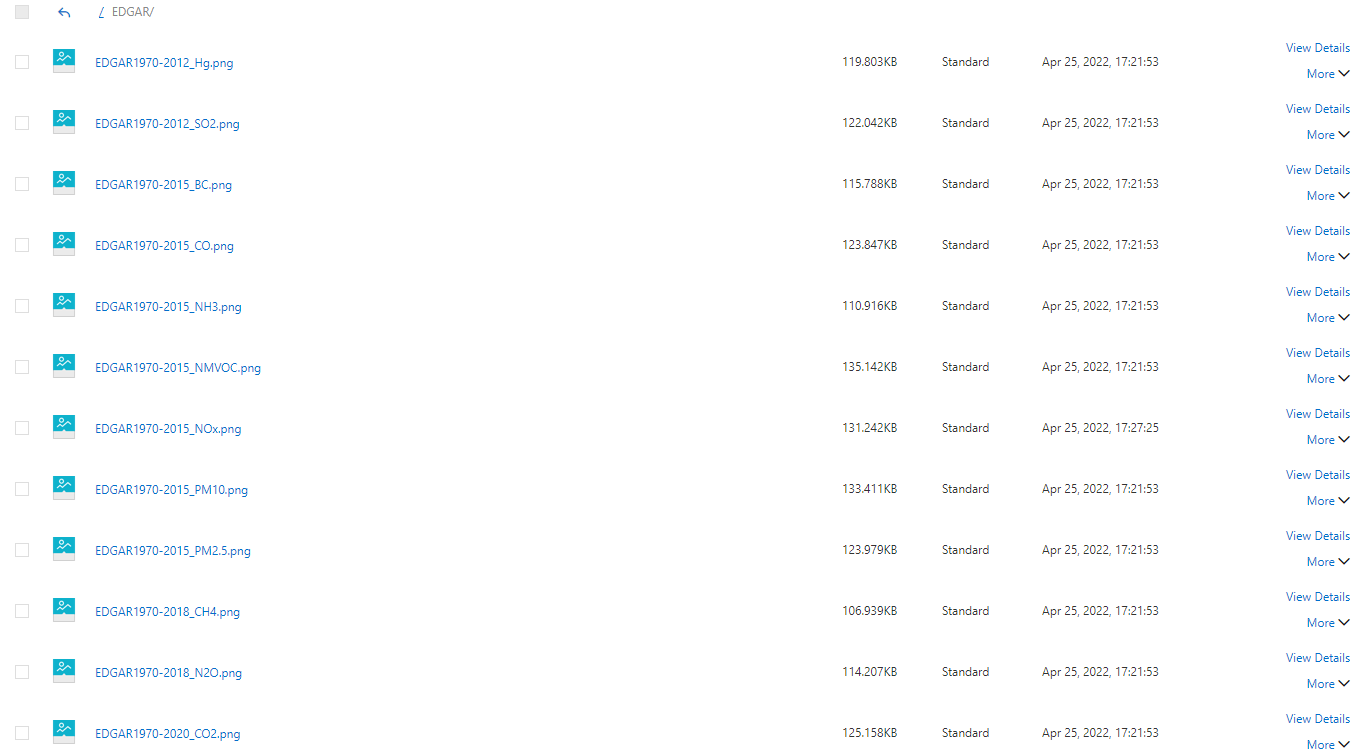


Figure 2 The list of partial uploaded source files from the EDGAR database


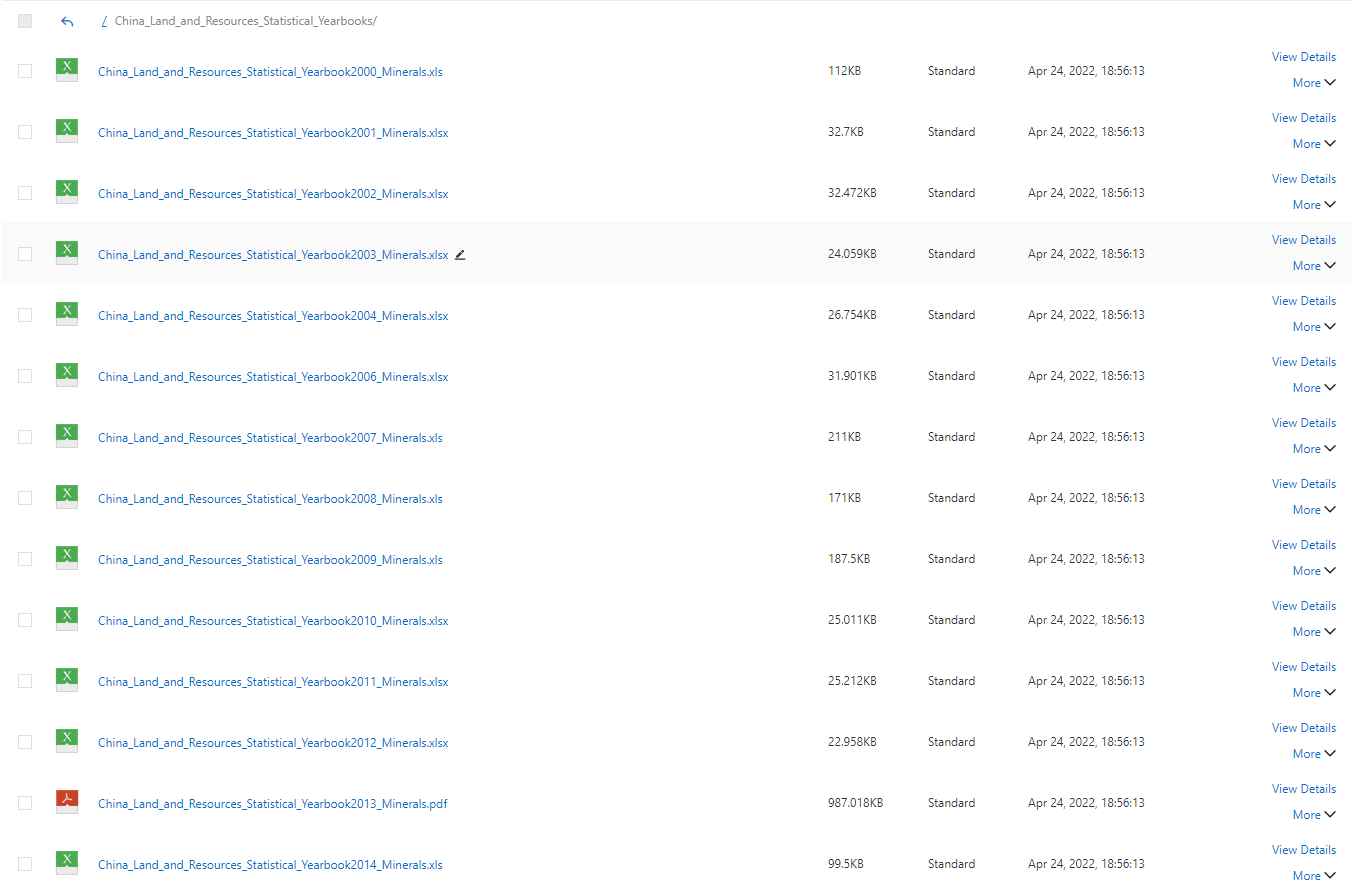


Figure 3 The list of partial uploaded source files of China Land and Resource Statistical Yearbooks

**Step 3: Link each data cell to its source file.**

In the data collection file, there are two sheets. One is the sheet (Figure 4) to record the location of all source files and their unique identification (ID), and the other (Figure 5) is the sheet to record statistics and their source ID. With these two sheets, the link between the statistics of each data cell and data sources is established.


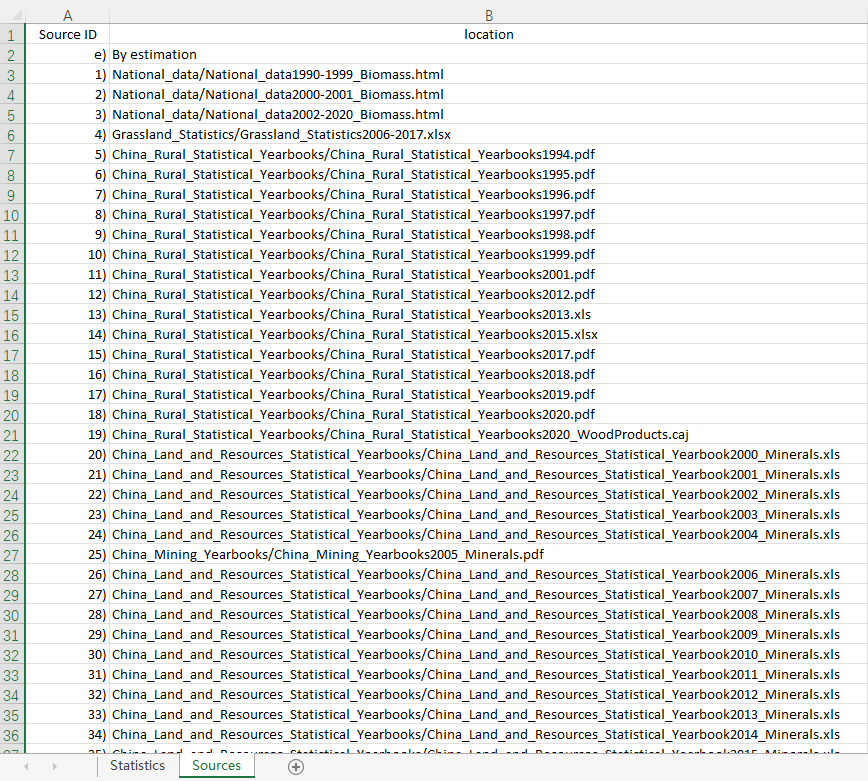


Figure 4 Location and ID of each source file in the data collection file (Sheet! Sources)


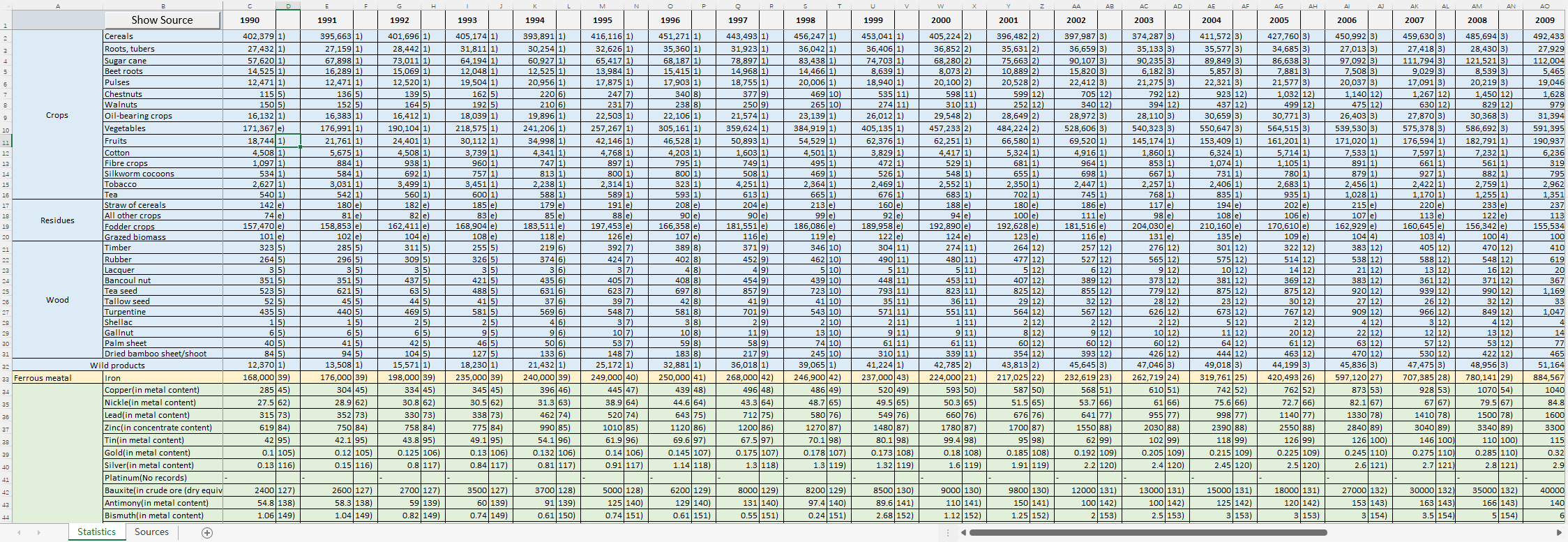


Figure 5 Each data cell and its source (ID) in the data collection file (Sheet! Statistics).

**Step 4: Use the tool to allow for quickly retrieving data source files.**

First (Figure 6), select the source ID with which the value needs to be checked. For example, as highlighted in red colour, the domestic extraction of fruits in 1990 is recorded in the National Data database and stored as the Source ID 1. Second, click the ‘Show Source’ bottom, and then its source file will be downloaded automatically. Last (Figure 7), the consistency between the collected data and the reported one could be quickly examined.


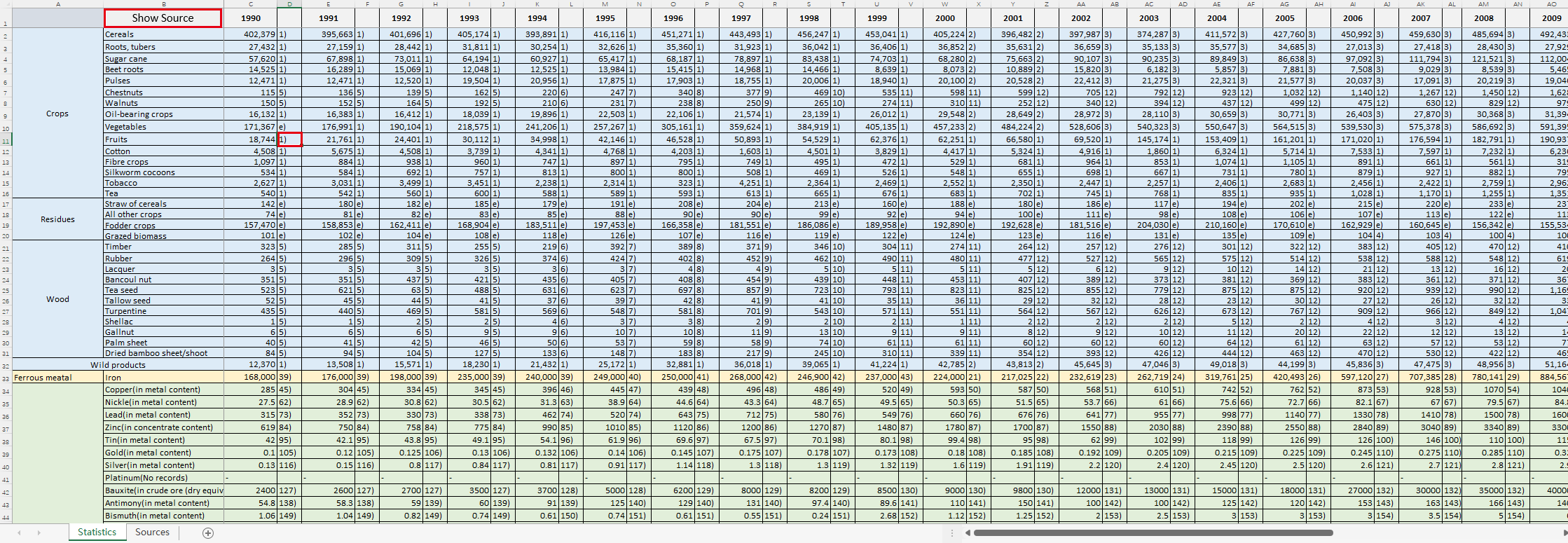


Figure 6 Select the source file.

Figure 7 Download the source file and compare the collected and the reported data.
